# *Vibrio parahaemolyticus* VtrA is a membrane-bound regulator and is activated via oligomerization

**DOI:** 10.1371/journal.pone.0187846

**Published:** 2017-11-17

**Authors:** Ryu Okada, Shigeaki Matsuda, Tetsuya Iida

**Affiliations:** Department of Bacterial Infections, Research Institute for Microbial Diseases, Osaka University, Suita, Osaka, Japan; Centre National de la Recherche Scientifique, Aix-Marseille Université, FRANCE

## Abstract

*Vibrio parahaemolyticus* is a Gram-negative pathogen that causes food-borne gastroenteritis. A major virulence determinant of the organism is a type III secretion system (T3SS2) encoded on a pathogenicity island, Vp-PAI. Vp-PAI gene expression is regulated by two transcriptional regulators, VtrA and VtrB, whose N-terminal regions share homology with an OmpR-family DNA-binding domain. VtrA activates the gene expression of VtrB, which in turn activates Vp-PAI gene expression; however, the mechanism of this transcriptional activation by VtrA is not well understood. In this study, we determined that VtrA is a membrane protein with a transmembrane (TM) domain, which was required for its transcriptional regulatory activity. Although the N-terminal region of VtrA alone is insufficient for its transcriptional regulatory activity, forced oligomerization using the leucine-zipper dimerization domain of yeast GCN4 conferred transcriptional regulatory activity and a greater affinity for the promoter region of *vtrB*. A ToxR-based assay demonstrated that VtrA oligomerizes *in vivo*. We also showed that bile, a host-derived activator of VtrA, induces the oligomerization of VtrA, which requires the C-terminal domain. The promoter region of *vtrB* contained repetitive T-rich DNA elements, which are important for *vtrB* transcriptional activation and are conserved among T3SS2-possessing *Vibrio* species. These findings propose that VtrA is active as oligomers, which may facilitate its N-terminus binding the target DNA, thus enhancing its transcriptional regulatory activity.

## Introduction

*Vibrio parahaemolyticus* is a Gram-negative halophilic bacterium that inhabits marine and estuarine environments [1]. The bacterium is a major food-borne pathogen that causes acute, seafood-associated gastroenteritis in humans worldwide [2–4]. Only a small subset of *V*. *parahaemolyticus* is associated with human infection. This association is closely related to the presence of a pathogenicity island, Vp-PAI, on the smaller of the two *V*. *parahaemolyticus* chromosomes [5–7]. The Vp-PAI region encodes one of the two type III secretion systems (T3SSs) of the organism, T3SS2 [5]. T3SSs are protein export systems that enable bacteria to secrete and translocate proteins, known as effectors, into the cytoplasm of host cells [8]. In addition to the epidemiological association, the intestinal pathology of *V*. *parahaemolyticus* is dependent on T3SS2 in several animal models [9–11]. Therefore, *V*. *parahaemolyticus* T3SS2 is thought to play an essential role in the enterotoxicity of the organism. The T3SS2 gene cluster is classified into two distinct phylogroups, T3SS2α and T3SS2β, which are distributed not only in *V*. *parahaemolyticus* but also in non-toxigenic *V*. *cholerae* and *V*. *mimicus* [12–14]. The T3SS of non-toxigenic *V*. *cholerae* is suggested to also be involved in the pathogenicity of the organism [12, 15].

The expression of Vp-PAI genes, including T3SS2 genes, is dependent on two transcriptional regulators, VtrA and VtrB, which are encoded in the Vp-PAI region [11, 16]. The N-terminal regions of VtrA and VtrB share homology with winged-helix-turn-helix (wHTH) DNA-binding domains of OmpR-family proteins including *Escherichia coli* OmpR and *V*. *cholerae* ToxR [16]. The N-terminal region of VtrA is capable of binding to the upstream region of *vtrB*, where VtrA positively regulates *vtrB* transcription. Therefore, it is thought that VtrA and VtrB constitute a regulatory cascade in which VtrA acts as an upstream regulator. A transcriptome analysis of *V*. *parahaemolyticus* has shown that the VtrA-VtrB regulatory cascade controls more than 60 genes, most of which are exclusively located in the Vp-PAI region. Vp-PAI gene expression is vigorously induced by bile in a *vtrA*-dependent manner, which contributes to the intestinal pathogenesis of *V*. *parahaemolyticus* [17]. Bile elevates the production of VtrB, while the protein level of VtrA is not affected. Therefore, it is likely that bile activates transcriptional regulatory activity of VtrA. A recent study reported that VtrC, a co-transcribed protein with VtrA, is required for the activation of T3SS2 by bile salts [18]. VtrC has been also shown to form a complex with the C-terminal domain of VtrA, which binds to bile salts. Thus, it has been proposed that the VtrA/VtrC complex senses bile salts to activate the DNA-binding domain of VtrA, but its mechanism is yet to be determined.

VtrA consists of 253 amino acids with a conserved DNA-binding region at the N-terminal side, described above. Despite a critical role of VtrA in the regulation of Vp-PAI genes and the pathogenesis of *V*. *parahaemolyticus*, its mechanism of transcriptional activation has been not well understood. Although VtrA was predicted to possess a putative transmembrane (TM) domain [16], it has not yet been determined whether VtrA is localized in the membrane. In this study, we experimentally determined that VtrA is a membrane protein with a TM domain. We show that VtrA forms oligomers via its TM and C-terminal domains, facilitating its N-terminal DNA-binding activity, and thereby enhancing its transcriptional regulatory activity. Furthermore, we found a T-rich DNA element in the *vtrB* promoter region that is conserved in other T3SS2-possessing *Vibrio* species and is essential for *vtrB* transcriptional activation by VtrA.

## Materials and methods

### Bacterial strains and plasmids

*V*. *parahaemolyticus* strain RIMD2210633 (a T3SS2α-positive clinical isolate) [5] was the wild-type strain in this study. *V*. *parahaemolyticus vtrA* deletion strain derived from the wild-type strain carries a stop codon at residue 30 and a deletion from nucleotides 88 to 471 in the *vtrA* gene [16]. To generate a Δ*vp0820* deletion mutant, a four-primer PCR technique with pYAK1, an R6K*ori* suicide vector containing *sacB*, was used as described previously [19]. *V*. *parahaemolyticus* strain TH3996 [13], and *V*. *cholerae* strains RIMD2214343 and RIMD2214428 [14] were obtained from the Laboratory for Culture Collection, Research Institute for Microbial Diseases, Osaka University. *E*. *coli* DH5α and SM10λ*pir* strains were used for general manipulation of plasmids and their mobilization into *V*. *parahaemolyticus*, respectively. *E*. *coli* MC4100 was used for β-galactosidase assays. The strains and plasmids used in this study are listed in S1 and S2 Tables, respectively.

### Bacteria fractionation

*V*. *parahaemolyticus* harboring pBAD-*vtrA*, pBAD-*vtrA-FLAG* or their derivatives were grown in Luria-Bertani (LB) media containing 0.5% NaCl in the presence of 0.01% (w/v) L-arabinose to an OD600 of 2.0. Then, the bacterial cells were harvested by centrifugation at 3,000 × *g* for 15 min. The pellets were re-suspended with 200 μl of the periplasting buffer (200 mM Tris-HCl at pH 7.5, 20% sucrose, 1 mM EDTA, and 20 mg/ml lysozyme) and incubated for 5 min at room temperature. The cells were osmotically shocked by the addition of 200 μl of ice-cold distilled water and incubated for 5 min on ice. After centrifugation at 12,000 × *g* for 2 min, the spheroplasts were treated with 500 U/ml benzonase for 5 min at room temperature, and sonicated to induce lysis. The sample was centrifuged twice at 12,000 × *g* for 2 min to remove unlysed cells. The supernatants were centrifuged at 100,000 × *g* for 1 h, and the resulting supernatants were collected as the cytoplasmic fraction. The membrane fractions were dissolved in SDS loading buffer. Each fraction was subjected to sodium dodecyl sulfate-polyacrylamide gel electrophoresis (SDS-PAGE) and immunoblot analysis using anti-HA monoclonal (Medical & Biological Laboratories, Nagoya, Japan), anti-FLAG monoclonal (Sigma-Aldrich, St. Louis, MO, USA), anti-DnaK and anti-OmpA antibodies.

### β-galactosidase assays

*V*. *parahaemolyticus* strains harboring pHRP309-derivative reporter plasmids were grown in LB broth with 0.5% NaCl at 37°C for 6 h. For the bile stimulation, *V*. *parahaemolyticus* cells were grown at 37°C for 3 h, and then incubated with 0.04% bile for another 3 h. *E*. *coli* strains harboring pHRP309-derivative reporter plasmids were grown in LB broth with 1% NaCl at 37°C for 3 h. In *V*. *parahaemolyticus* and *E*. *coli* strains carrying pBAD-*vtrA* or its derivatives, the expression of both HA-VtrA and its truncated forms was induced by addition of L-arabinose to a final concentration of 0.001% (w/v). The β-galactosidase activity of cell lysates was measured using Miller's method with the substrate *o*-nitrophenyl-β-D-galactopyranoside [20].

### A ToxR-based transcriptional reporter assay

A ToxR-based transcriptional reporter assay was performed on *V*. *parahaemolyticus* Δ*vtrA* Δ*vp0820* carrying pHRP309-P_*ompU*_ and either pBAD-*toxR*, pBAD-*toxR*^*N*^ or pBAD-*toxR*^*N*^-*vtrA*^*TM-C*^. The resulting strains were grown in LB media with 0.5% NaCl in the presence of 0.001% (w/v) L-arabinose at 37°C for 6 h. For the bile stimulation, *V*. *parahaemolyticus* cells were grown at 37°C for 3 h, and then incubated with 0.04% bile for another 3 h. The β-galactosidase activity of cell lysates was measured as described above.

### Protein expression, purification and cross-linking

The 6×Histidine-tagged N-terminal region of VtrA (His-VtrA^N^: aa 1–133) or its leucine-zipper dimerizing domain (ZIP)-fused proteins were expressed and purified from *E*. *coli* BL21(DE3) harboring pET28a expression plasmids containing the genes for VtrA^N^ or its ZIP-fusions as described previously [21]. For cross-linking, 1 μg of each purified protein was incubated with 7.5 mg/ml dimethyl adipimidate (DMA) in phosphate-buffered saline containing 50 mM triethanolamine for 1 h at room temperature. Then, the protein was mixed with an equal volume of 2× SDS-loading buffer and boiled for 5 min. These samples were subjected to SDS-PAGE, followed by staining with Coomassie brilliant blue (CBB).

### Electrophoretic mobility shift assays

The DNA fragments corresponding to the 284-bp upstream region of *vtrB* were amplified by PCR using 5’-biotinylated primers. The PCR products were separated by agarose gel electrophoresis and purified using the QIAquick Gel Extraction Kit (Qiagen, Valencia, CA). This product was used for the biotinylated DNA probe. Increased concentrations of His-VtrA^N^ or ZIP-fused VtrA^N^ proteins were incubated with 4 nM biotinylated DNA probe in the reaction buffer (10 mM Tris-HCl at pH 7.5, 100 mM KCl, 1 mM dithiothreitol, 1 mM EDTA, 0.2% Nonidet P-40, 10% glycerol and 100 ng/ml bovine serum albumin) for 30 min at room temperature. Reaction mixtures were then separated by 6% non-denaturing polyacrylamide gel electrophoresis in TGE buffer (25 mM Tris, 190 mM glycine and 1 mM EDTA) containing 100 mM KCl at 4°C. The DNA probe was electroblotted to a positively charged nylon membrane, UV cross-linked, probed with horseradish peroxidase (HRP)-conjugated streptavidin and developed using the ECL Western blotting kit (GE Healthcare, Buckinghamshire, UK) according to the manufacturer's instructions.

### Cytotoxicity assays

*V*. *parahaemolyticus* strains were grown in LB medium supplemented with 0.5% NaCl in the presence or absence of 0.04% bile for 3 h and then used for infections. HeLa cells were seeded at 1 × 10^4^ cells per well in 96-well plates and grown for 48 h. The cells were then infected with *V*. *parahaemolyticus* at a multiplicity of infection of 10 in Dulbecco’s modified Eagle’s medium (Sigma-Aldrich) without serum for 4.5 h. The cytotoxicity assay was performed using the CytoTox96 Non-Radioactive Cytotoxicity Assay Kit (Promega, Madison, WI) as previously described [19, 21].

### Mapping of transcriptional start site by 5′ random amplification of cDNA ends (5′-RACE)

Total RNA from *V*. *parahaemolyticus* was isolated using an RNeasy Mini Kit (Qiagen) according to the manufacturer's instructions. The 5’ end of the *vtrB* transcript was determined using the SMARTer RACE 5’/3’ Kit (Takara Bio, Shiga, Japan) according to the manufacturer's instructions. Nucleotide sequencing was performed with the BigDye v3.1 cycle sequencing kit (Applied Biosystems, Foster City, CA) and the ABI PRISM 3100 genetic analyzer (Applied Biosystems). The putative −35 and −10 promoter elements were predicted using the software GENETYX ver. 11 (GENETYXS, Tokyo, Japan).

### Statistical analysis

Statistical analysis was performed using the two-tailed Student’s *t*-test or a one-way ANOVA followed by Dunnett’s multiple comparison test. A p-value < 0.05 was considered statistically significant.

## Results

### VtrA is a transmembrane protein localized in the membrane via its TM domain

**Fig 1:**
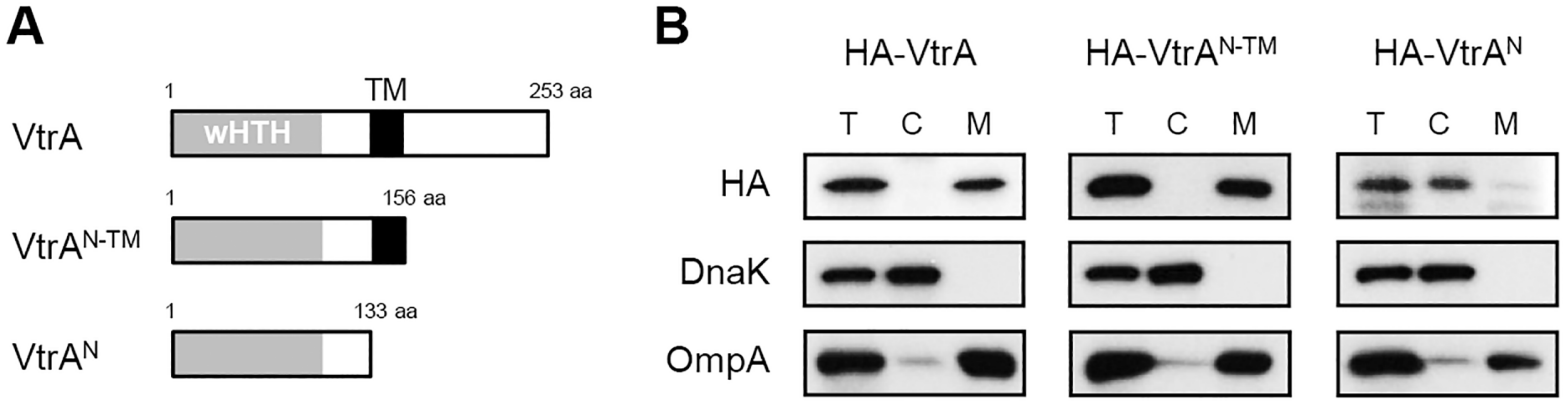
VtrA is a membrane-bound protein with a TM domain. (A) Schematic representations of full-length and truncated VtrA. wHTH, winged-helix-turn-helix domain; TM, putative transmembrane domain. (B) Subcellular localization of VtrA in *V*. *parahaemolyticus*. *V*. *parahaemolyticus* expressing HA-VtrA and its truncated derivatives were fractionated into cytosol (C) and membrane (M) fractions. Each of fractions and total cell lysates (T) were subjected to immunoblot analysis for HA to detect VtrA and its truncated derivatives. DnaK and OmpA were detected as controls for the cytosol and membrane, respectively.

We first predicted the subcellular localization of VtrA *in silico* using a topological analysis program TMHMM v. 2.0 (http://www.cbs.dtu.dk/services/TMHMM/). VtrA was predicted as a bitopic membrane protein with its N-terminus (aa 1–133) in the cytosol, a single TM region located between amino acid residues (aa) 134 and 156, and its C-terminus (aa 157–253) in the periplasm (Fig 1A). To verify whether VtrA was located in the membrane, a derivative of the arabinose-inducible plasmid, pBAD18-Cm, encoding an N-terminal, hemagglutinin (HA)-tagged VtrA (pBAD-*vtrA*) was introduced into a *V*. *parahaemolyticus vtrA* deletion strain (Δ*vtrA*). The resulting strain Δ*vtrA*/pBAD-*vtrA* was fractionated into cytosolic and membrane fractions. HA-tagged VtrA was detected in the membrane fractions, indicating that VtrA is localized in the membrane of *V*. *parahaemolyticus* (Fig 1B). Because VtrA possesses a putative TM region, we next investigated whether this region is involved in the membrane targeting of VtrA. We constructed two pBAD-*vtrA* derivatives encoding truncated forms of the HA-tagged protein. The first, pBAD-*vtrA*^*N*^, encodes only the N-terminal cytosolic domain of VtrA (aa 1–133; VtrA^N^). The second, pBAD-*vtrA*^*N-TM*^, encodes the N-terminal cytosolic domain and putative TM region but lacks the C-terminal putative periplasmic region of VtrA (aa 1–156; VtrA^N-TM^). Subcellular fractionation showed that VtrA^N-TM^ was still localized in the membrane fractions, whereas VtrA^N^ was enriched in the cytosolic fractions (Fig 1B). Similar results were observed in *V*. *parahaemolyticus* cells expressing C-terminal 3×FLAG-tagged VtrA and its truncated forms (S1 Fig). Taken together, these results indicate that VtrA is a membrane protein whose localization to the membrane depends on the TM domain.

### N-terminal DNA-binding domain of VtrA alone is insufficient for transcriptional regulatory activity

**Fig 2:**
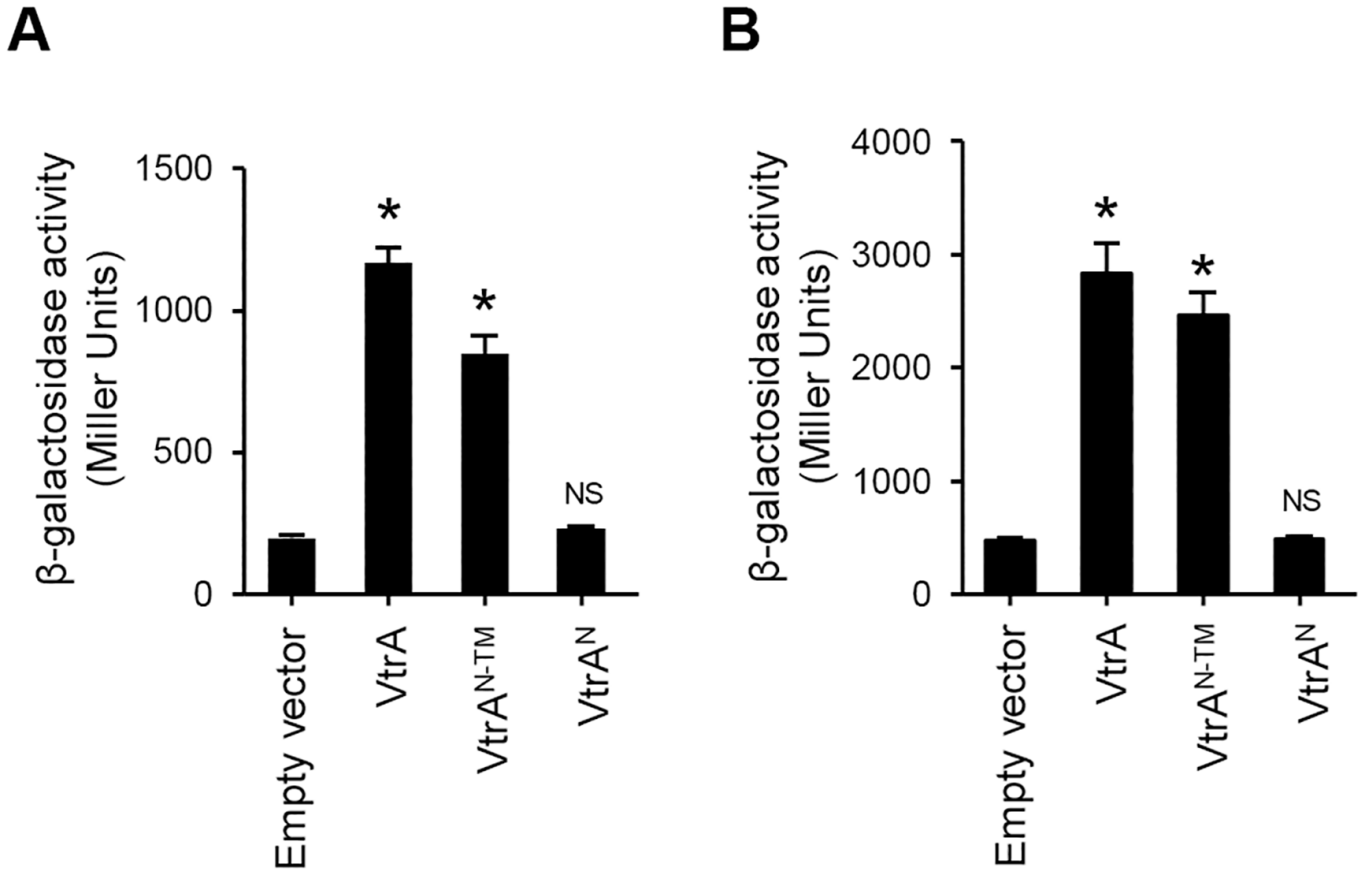
Transcription of *vtrB* is activated by VtrA and VtrA^N-TM^ but not VtrA^N^. β-galactosidase activity from the P_*vtrB*_-*lacZ* transcriptional reporter of *V*. *parahaemolyticus* Δ*vtrA* (A) and *E*. *coli* MC4100 (B) upon expression of VtrA and its truncated derivatives. The values represent the mean ± standard deviations (SD) for a minimum of three independent experiments. *, p < 0.01; NS, not significant, compared with empty vector, determined by one-way ANOVA followed by Dunnett’s multiple comparison test.

In a previous report, it has been shown that the N-terminal cytosolic region of VtrA can bind upstream regions of *vtrB*, activating its transcription [16]. Using a *lacZ* reporter gene assay, we next investigated whether the N-terminal region of VtrA was sufficient to activate *vtrB* transcription. A pHRP309-derived *lacZ* transcriptional fusion reporter, pHRP309-P_*vtrB*_ [16], containing 284-bp upstream promoter region of *vtrB* (P_*vtrB*_), was introduced into *V*. *parahaemolyticus* strains Δ*vtrA*/pBAD-*vtrA*, Δ*vtrA*/pBAD-*vtrA*^*N*^ and Δ*vtrA*/pBAD-*vtrA*^*N-TM*^, and *vtrB* transcription was evaluated by measuring the β-galactosidase activity. P_*vtrB*_*-lacZ* transcription was induced in Δ*vtrA*/pBAD-*vtrA*^*N-TM*^ as well as in Δ*vtrA*/pBAD-*vtrA*, but not in Δ*vtrA*/pBAD-*vtrA*^*N*^ (Fig 2A). Additionally, to avoid the potential effects of other factors in *V*. *parahaemolyticus*, we evaluated the VtrA-mediated transcriptional activation by heterologous expression in *E*. *coli*. VtrA and its truncated forms were expressed in *E*. *coli* MC4100 cells harboring pHRP309-P_*vtrB*_ and were evaluated for their ability to activate *vtrB* transcription. P_*vtrB*_*-lacZ* expression was significantly induced in an *E*. *coli* strain expressing full-length VtrA, compared with a strain bearing an empty vector (Fig 2B). The heterologous expression of VtrA^N-TM^ in *E*. *coli* likewise induced the *lacZ* expression, whereas *E*. *coli* expressing VtrA^N^ showed no increase in the *lacZ* expression. Together, these data demonstrated that VtrA^N^ alone is insufficient for the transcriptional activation of *vtrB*, suggesting that the other domains of VtrA are also involved.

### Oligomerization of the N-terminal cytosolic DNA-binding domain is important for the transcriptional activation by VtrA

Many bacterial transcription factors function as oligomers in their activated states [22]. These include various membrane-bound transcriptional regulators whose cytosolic DNA-binding domains oligomerize through interactions between their TM and/or periplasmic domains [23–27]. Given that the transcriptional regulatory activity of VtrA is abolished by the deletion of its TM and C-terminal domains, we hypothesized that VtrA activation might also be mediated by oligomerization through these domains. To explore this hypothesis, we constructed an additional pBAD-*vtrA* derivative encoding a VtrA variant in which the TM domain was replaced with a TM domain composed of polyleucine (pBAD-*vtrA*-PL; S2A Fig). The TM domain composed of polyleucine is known to have a less tendency to oligomerize in *E*. *coli* [27, 28]. VtrA-PL was expressed in *E*. *coli* MC4100 cells harboring pHRP309-P_*vtrB*_ and evaluated for its ability to activate *vtrB* transcription. The expression of VtrA-PL did not significantly induce P_*vtrB*_*-lacZ* expression, in contrast to the expression of VtrA (S2B Fig). Thus, this result suggested that the TM domain plays major roles in transcriptional activation of VtrA, through its oligomerization.

**Fig 3:**
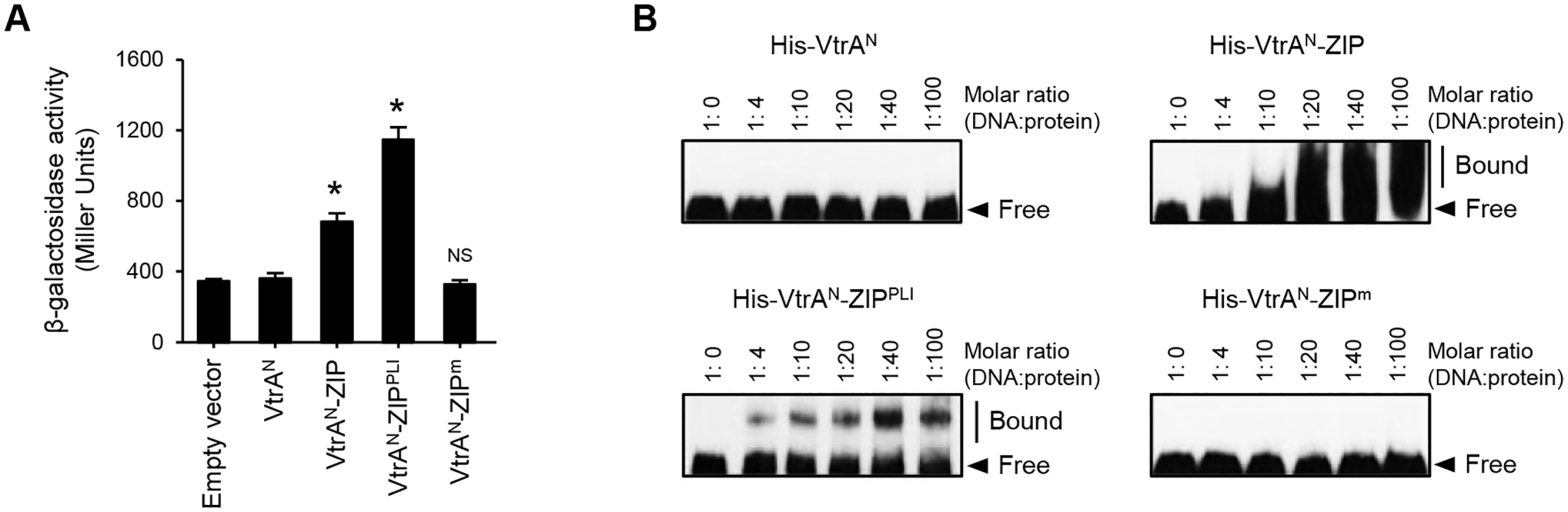
Oligomerization of the N-terminal DNA-binding region of VtrA is important for the transcriptional regulatory activity of VtrA. (A) β-galactosidase activity from P_*vtrB*_-*lacZ* transcriptional reporter of *E*. *coli* MC4100 upon expression of VtrA^N^ and its ZIP-fusions. The values represent the mean ± SD for a minimum of three independent experiments. *, p < 0.01; NS, not significant, compared with VtrA^N^ by one-way ANOVA followed by Dunnett’s multiple comparison test. (B) Electrophoretic mobility shift assays using VtrA^N^ and its ZIP fusions. Increased concentrations (0, 16, 40, 80, 160 and 400 nM) of each protein were incubated with 4 nM biotinylated DNA probe corresponding to 284-bp upstream region of *vtrB*, and then subjected to native PAGE. The biotinylated probe was transferred to a nylon membrane, UV cross-linked, and detected using HRP-conjugated streptavidin.

If the transcriptional regulatory activity of VtrA is activated via oligomerization, the forced oligomerization of VtrA^N^ should exhibit transcriptional activation of *vtrB*. To examine this hypothesis, we used the ZIP domain of *Saccharomyces cerevisiae* transcriptional activator GCN4 [29] to force oligomerization of VtrA^N^. VtrA^N^ was fused to a wild-type ZIP, a ZIP tetrameric mutant domain (ZIP^PLI^), and a ZIP monomeric mutant (ZIP^m^), yielding dimeric, tetrameric or monomeric VtrA^N^. We confirmed the oligomeric properties of VtrA^N^-ZIP fusion proteins. The 6×Histidine (His)-tagged VtrA^N^, VtrA^N^-ZIP, VtrA^N^-ZIP^PLI^ and VtrA^N^-ZIP^m^ proteins were purified, chemically crosslinked, and visualized by CBB staining of SDS-PAGE gels (S3 Fig). Without crosslinking, all ZIP-fused VtrA^N^ migrated at ~26 kDa, consistent with the molecular weight of the monomer. Cross-linked VtrA^N^-ZIP and VtrA^N^-ZIP^PLI^ migrated at ~50 kDa and ~110 kDa, respectively. By contrast, cross-linked VtrA^N^-ZIP^m^ migrated at ~26 kDa, indicating its monomeric structure. Thus, these results confirmed that these fusion proteins form the desired oligomeric states. We further found that the expression of the VtrA^N^-ZIP fusion protein (VtrA^N^-ZIP) in *E*. *coli* MC4100 cells harboring pHRP309-P_*vtrB*_ induced significant *lacZ* expression, in contrast to the expression of VtrA^N^ alone (Fig 3A). Interestingly, the expression of VtrA^N^ fused to tetrameric ZIP^PLI^ (VtrA^N^-ZIP^PLI^) showed increased expression of *lacZ*, compared to that of dimeric VtrA^N^-ZIP. By contrast, VtrA^N^ fused to monomeric ZIP^m^ (VtrA^N^-ZIP^m^) failed to induce the *lacZ* expression. Taken together, the forced oligomerization of VtrA^N^ conferred the expected transcriptional activation of *vtrB*, suggesting that the oligomerization of the cytosolic DNA-binding domain is important for the transcriptional regulatory activity of VtrA.

### Forced oligomerization of VtrA enhances its DNA-binding activity

To investigate the mechanism underlying the oligomerization-mediated activation of VtrA, we examined the DNA-binding activity of VtrA^N^-ZIP fusion proteins using an electrophoretic mobility shift assay (EMSA). DNA fragments corresponding to the 284-bp upstream region of *vtrB* were incubated with His-VtrA^N^ and its ZIP-fusions at various DNA/protein molar ratios (from 1: 0 to 1: 100), and then subjected to native gel electrophoresis. Neither His-VtrA^N^ nor His-VtrA^N^-ZIP^m^ (monomeric) induced a shift of the DNA probe at a molar ratio of 1:100 (Fig 3B). Note that the binding of His-VtrA^N^ to the probe was observed at higher concentrations of His-VtrA^N^ (S4 Fig). By contrast, His-VtrA^N^-ZIP induced the mobility shift even at a molar ratio of 1:10. Furthermore, His-VtrA^N^-ZIP^PLI^ bound to the probe at lower concentration than His-VtrA^N^-ZIP. Therefore, the forced oligomerization of VtrA^N^ conferred a greater affinity for the promoter region of *vtrB*. These results suggest that the oligomerization of VtrA facilitates its DNA-binding activity, thus enhancing the transcriptional regulatory activity of VtrA.

### VtrA forms oligomers *in vivo*

**Fig 4:**
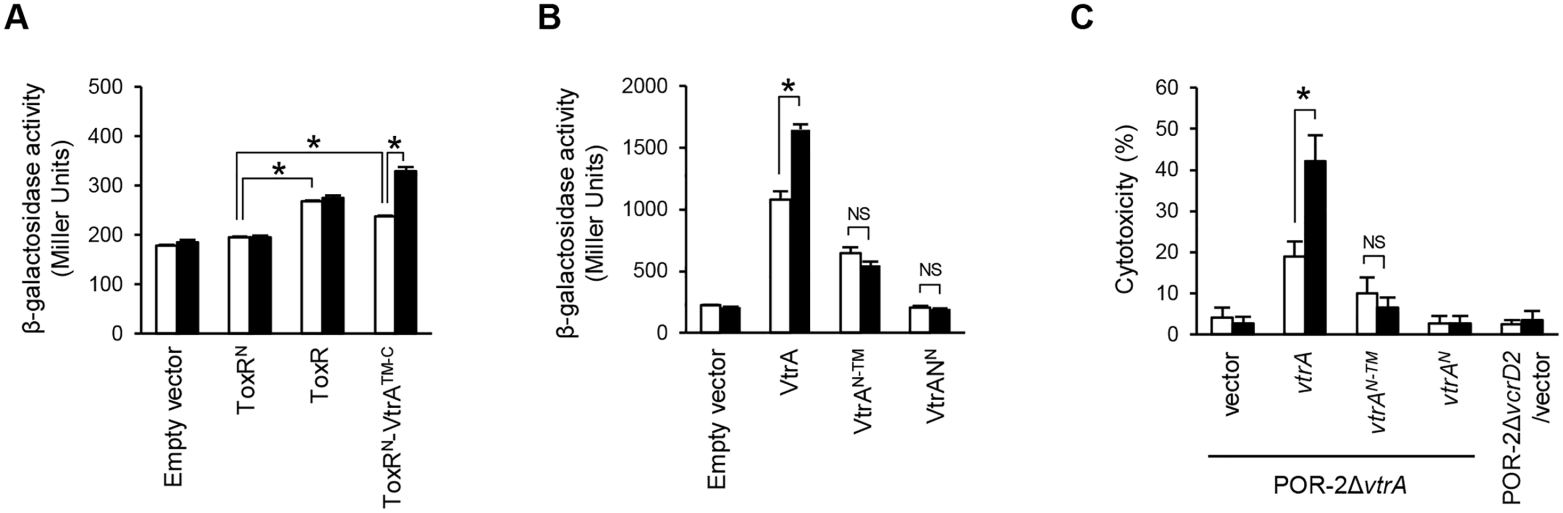
Oligomerization of VtrA in *V*. *parahaemolyticus* during its active states. (A) β-galactosidase activity from the P_*ompU*_-*lacZ* transcriptional reporter of *V*. *parahaemolyticus* Δ*vtrA* Δ*vp0820* upon expression of ToxR, N-terminal domain of ToxR (ToxR^N^) and ToxR^N^-fused VtrA^TM-C^, in the absence (white bar) or presence of 0.04% bile (black bar). *, p < 0.01; NS, by Student’s *t*-test. (B) β-galactosidase activity from the P_*vtrB*_-*lacZ* transcriptional reporter of *V*. *parahaemolyticus* Δ*vtrA* upon expression of VtrA and its truncated derivatives, in the absence (white bar) or presence of 0.04% bile (black bar). *, p < 0.01; NS, not significant, by Student’s *t*-test. (C) HeLa cells were infected with the indicated stains of *V*. *parahaemolyticus* unstimulated (white bar) or stimulated with bile (black bar) for 4.5 h. The cytotoxicity was evaluated using the lactate dehydrogenase release assay. *, p < 0.01; NS, not significant, by Student’s *t*-test. The values represent the mean ± SD for a minimum of three independent experiments (A—C).

To examine whether VtrA forms oligomers in *V*. *parahaemolyticus*, we employed a ToxR-based transcriptional reporter assay [30]. *V*. *cholerae* ToxR is a membrane-spanning transcriptional regulator that activates the transcription of its target genes including *ctx* and *ompU*, in a manner dependent on the dimerization of its cytoplasmic DNA-binding domain [31, 32]. We generated a chimeric construct in which the TM and C-terminal putative periplasmic domains of VtrA (aa 134–253; VtrA^TM-C^) were fused to the DNA-binding domain of *V*. *cholerae* ToxR (ToxR^N^), and evaluated whether the ToxR^N^-VtrA^TM-C^ fusion could activate the *ompU* promoter (P_*ompU*_) in *V*. *parahaemolyticus*. *V*. *parahaemolyticus* has a ToxR homologue, VP0820, which bears homology with *V*. *cholerae* ToxR [33]. To exclude the potential effects of VP0820 on our assay, a deletion strain in the Δ*vtrA* background was constructed (Δ*vtrA* Δ*vp0820*) and used for the P_*ompU*_-*lacZ* reporter assay. Using a *V*. *parahaemolyticus* Δ*vtrA* Δ*vp0820* strain carrying a P_*ompU*_-*lacZ* reporter plasmid, we confirmed that the expression of full-length ToxR increased P_*ompU*_-*lacZ* transcription, in contrast to that of ToxR^N^ (Fig 4A). The expression of the ToxR^N^-VtrA^TM-C^ fusion showed increased P_*ompU*_-*lacZ* transcription, thus indicating the oligomerization of VtrA^TM-C^ *in vivo*.

### Bile induces oligomerization of VtrA, which is dependent on the C-terminal domain

Bile is a host-derived factor that induces Vp-PAI gene expression in a *vtrA*-dependent manner [17]. The inducing activity of bile results in the elevation of *vtrB* transcription without affecting expression levels of VtrA, suggesting that the transcriptional regulatory activity of VtrA is activated by bile. It was reported that the C-terminal region of VtrA forms a complex with VtrC, which senses bile acids to activate the DNA-binding domain of VtrA through an undefined mechanism [18]. We observed that the bile treatment elevated P_*vtrB*_*-lacZ* transcription in *V*. *parahaemolyticus* Δ*vtrA*/pBAD-*vtrA*, but not in *V*. *parahaemolyticus* Δ*vtrA*/pBAD-*vtrA*^*N-TM*^ (Fig 4B), supporting that the C-terminal domain of VtrA is required for the bile-mediated activation of VtrA. Because we observed that transcriptional regulatory activity of VtrA^N^ is activated via oligomerization (Fig 3), we hypothesized that bile induces oligomerization of VtrA, resulting in the induction of VtrA transcriptional regulatory activity. To test this hypothesis, we examined the effect of bile on the transcriptional activation by ToxR^N^-VtrA^TM-C^ in *V*. *parahaemolyticus* Δ*vtrA* Δ*vp0820* carrying a P_*ompU*_-*lacZ* reporter plasmid. As expected, the treatment of bile enhanced the P_*ompU*_-*lacZ* transcription by a ToxR^N^-VtrA^TM-C^ fusion (Fig 4A). These results suggest that bile activates VtrA by enhancing oligomerization of VtrA in *V*. *parahaemolyticus*.

One virulence characteristic of *V*. *parahaemolyticus* is its T3SS2-dependent cytotoxicity in eukaryotic cultured cells. To address whether the oligomeric property of VtrA is correlated with the T3SS2-dependent cytotoxicity during infection, we evaluated the cytotoxicity of *V*. *parahaemolyticus* expressing truncated forms of VtrA. In HeLa cells, a *V*. *parahaemolyticus* Δ*vtrA* deletion strain derived from a T3SS1-defective strain POR-2 (POR-2Δ*vtrA*) did not exhibit cytotoxicity, which is similar to previous results observed in Caco-2 cells (Fig 4C) [19]. Complementation with plasmid-borne *vtrA* in a Δ*vtrA* strain (POR-2Δ*vtrA*/pBAD-*vtrA*) reconstituted the cytotoxicity, whereas complementation with *vtrA*^*N*^ (POR-2Δ*vtrA*/pBAD-*vtrA*^*N*^) did not. Bile is known to stimulate the T3SS2-dependent cytotoxicity [17]. POR-2Δ*vtrA*/pBAD-*vtrA* exhibited elevated cytotoxicity when stimulated with bile, which reflects the bile-induced T3SS2-related gene expression. In contrast, complementation with *vtrA*^*N-TM*^ in the Δ*vtrA* strain (POR-2Δ*vtrA*/ pBAD-*vtrA*^*N-TM*^) did not exhibit significant cytotoxicity regardless of the stimulation with bile. Collectively, these observations indicate that the C-terminal domain of VtrA plays an essential role in T3SS2 gene expression in response to bile.

### T-rich DNA elements in the *vtrB* promoter region are important for *vtrB* transcriptional activation by VtrA and conserved among T3SS2-possessing *Vibrio* species

**Fig 5:**
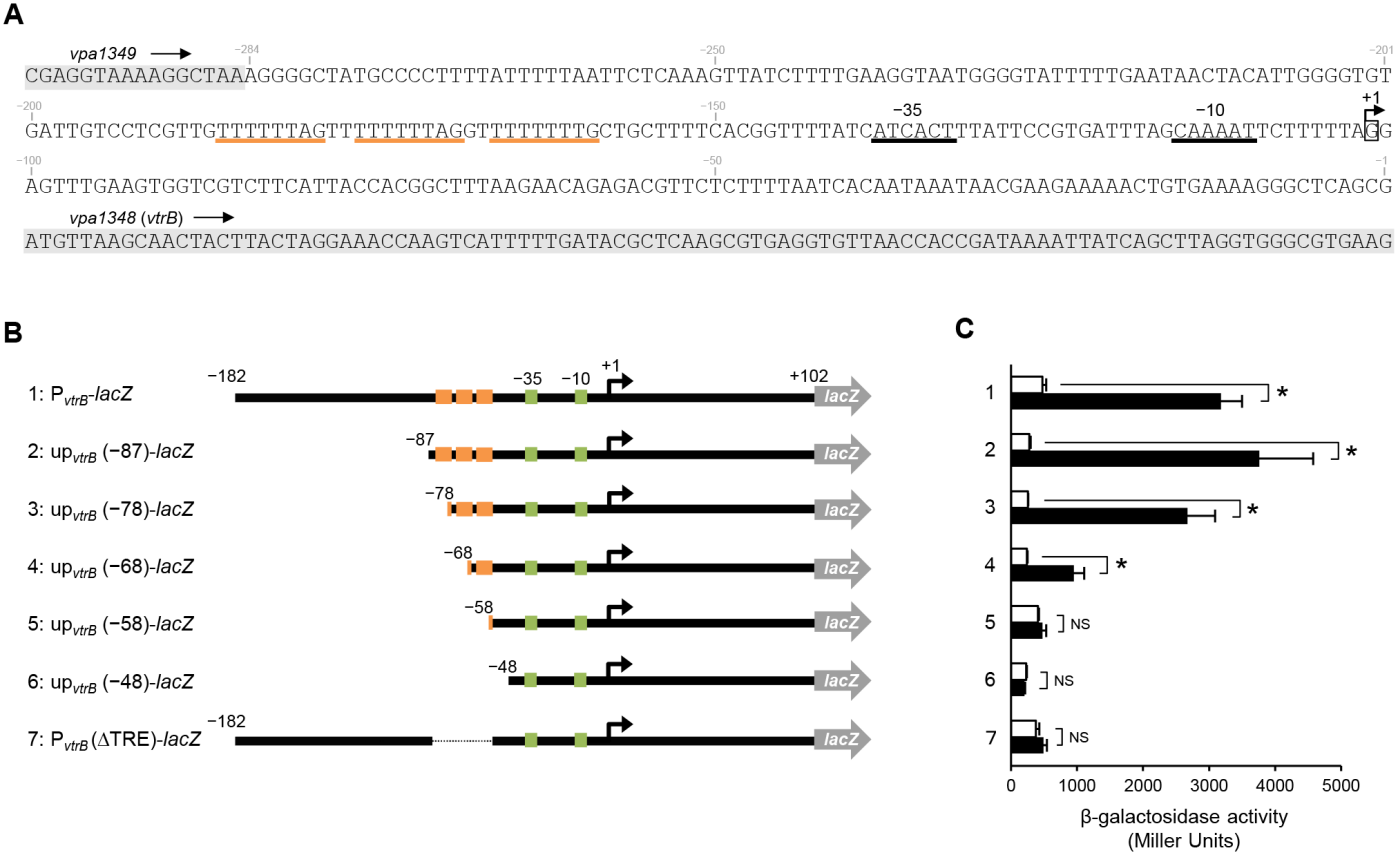
T-rich elements in the *vtrB* promoter region are required for transcriptional activation by VtrA. (A) The nucleotide sequence of the *vtrB* promoter region. The transcriptional start site of the *vtrB*, determined by 5’-RACE, is indicated as +1. Putative −35 and −10 elements are underlined. Repetitive T-rich elements are underlined in orange. Gray shading indicates the regions of coding sequence of *vpa1349* and *vpa1348*. (B) Schematics representing *lacZ* fusion reporters of the upstream promoter region of *vtrB* (P_*vtrB*_) and its truncated forms (up_*vtrB*_). Putative −35 and −10 promoter elements are shown in green. T-rich elements are indicated in orange. (C) β-galactosidase activity from P_*vtrB*_-*lacZ* and up_*vtrB*_-*lacZ* transcriptional reporters of *V*. *parahaemolyticus* Δ*vtrA* carrying an empty vector (white bar) or a VtrA expression plasmid (black bar). The values represent the mean ± SD for a minimum of three independent experiments. *, p < 0.01; NS, not significant, by Student’s *t*-test.

To analyze how VtrA recognizes the promoter region of *vtrB*, we mapped the transcriptional start site of *vtrB* using 5′-RACE and identified that the +1 nucleotide of the *vtrB* mRNA is 102 bp upstream of the ATG start codon (Fig 5A). The presence of putative −35 and −10 promoter elements was predicted in the upstream region. In addition, we found an 8-bp T-rich DNA element (5’-TTTTTTWG-3’) near the −35 promoter element, which was located 57 bp upstream of the transcription start site and repeated three times. To investigate whether the repetitive elements are involved in transcriptional activation of *vtrB* by VtrA, we prepared a series of truncated versions of the upstream region of *vtrB* (Fig 5B). These fragments were inserted into the pHRP309 plasmid, yielding up_*vtrB*_-*lacZ* transcriptional fusion reporter plasmids. Using these *lacZ* fusion reporters, we evaluated the transcriptional activation by VtrA in *E*. *coli* (Fig 5C). VtrA induced *lacZ* transcription from up_*vtrB*_-*lacZ* fusion reporters containing more than one T-rich element. The level of transcription is dependent on the number of T-rich elements, and the maximum *lacZ* transcription was observed in *E*. *coli* bearing the up_*vtrB*_ (−87)-*lacZ* fusion, which contains all of three T-rich elements. By contrast, no significant *lacZ* transcription was observed from the up_*vtrB*_ (−58)-*lacZ* and up_*vtrB*_ (−48)-*lacZ* reporters, both of which contain no T-rich element. Similarly, in *E*. *coli* bearing the P_*vtrB*_ (ΔTRE)-*lacZ* fusion, in which T-rich elements were deleted, VtrA did not significantly induce *lacZ* transcription. Thus, the T-rich DNA element is essential for the transcriptional activation of *vtrB* by VtrA.

*Vibrio* T3SS2 gene clusters are classified into two phylogroups, T3SS2α and T3SS2β, and are distributed among *Vibrio* species, including *V*. *parahaemolyticus* and non-toxigenic *V*. *cholerae* [13, 14]. Comparison of the amino acid sequences of VtrA from *V*. *parahaemolyticus* RIMD2210633 (T3SS2α-type), *V*. *parahaemolyticus* TH3996 (T3SS2β-type), *V*. *cholerae* RIMD2214343 (T3SS2α-type) and *V*. *cholerae* RIMD2214428 (T3SS2β-type) showed that the VtrA of *V*. *parahaemolyticus* RIMD2210633 is more closely related to the VtrA of *V*. *cholerae* RIMD2214343 than to the VtrAs of *V*. *parahaemolyticus* TH3996 and *V*. *cholerae* RIMD2214428. We aligned the nucleotide sequences of the upstream region of *vtrB* from these four strains and found that all four strains contain the repetitive T-rich elements (S5A Fig). To examine whether VtrA from *V*. *parahaemolyticus* RIMD2210633 recognizes the promoter region of *vtrB* from other T3SS2-possessing vibrios, we evaluated the transcriptional regulatory activity of VtrA toward *vtrB* of *V*. *parahaemolyticus* TH3996, *V*. *cholerae* RIMD2214343 and *V*. *cholerae* RIMD2214428. The upstream promoter regions of *vtrB* (P_*vtrB*_) from these strains were inserted into pHRP309 plasmid, and the resulting P_*vtrB*_-*lacZ* reporter plasmids were introduced into *E*. *coli* MC4100 harboring pBAD-*vtrA*. As shown in S5B Fig, VtrA from *V*. *parahaemolyticus* RIMD2210633 induced *lacZ* transcription from all P_*vtrB*_-*lacZ* fusion reporters. Thus, these results suggested that VtrA recognizes the promoter structure of *vtrB* which is conserved among T3SS2-possessing *Vibrio* species.

## Discussion

In the present study, we experimentally determined that VtrA is a membrane protein with a central TM domain. VtrA also contains an N-terminal wHTH domain and C-terminal putative periplasmic domain, thus assuming a bitopic membrane protein. VtrA is a transcriptional regulator that plays a central role in the expression of the Vp-PAI genes of *V*. *parahaemolyticus*. The N-terminal wHTH domain of VtrA has homology with the conserved DNA binding domains of OmpR-family proteins. The OmpR-family contains many members that function as transcriptional regulators, including *E*. *coli* OmpR, *V*. *cholerae* ToxR and *Salmonella Typhimurium* HilA. *E*. *coli* OmpR is a response regulator of the EnvZ/OmpR two-component system with its DNA-binding domain at the C-terminal side [34]. On the other hand, *V*. *cholerae* ToxR and *Salmonella Typhimurium* HilA contain their DNA-binding domains on their N-terminal sides, which is similar to VtrA. *V*. *cholerae* ToxR is a single-span membrane protein with a TM domain and periplasmic domain at its C-terminus, whereas *Salmonella Typhimurium* HilA lacks a membrane-spanning region [35]. Given the defined localization of VtrA in the membrane and the presence of the TM domain, VtrA is a membrane-bound regulator belonging to a group of ToxR-like protein.

ToxR-like proteins such as *V*. *cholerae* ToxR, *V*. *cholerae* TcpP and *E*. *coli* CadC are generally active as dimers [23, 25, 27]. We showed that VtrA also forms oligomers through its TM and C-terminal domains *in vivo* using a ToxR-based assay. Our β-galactosidase assays using VtrA truncated variants and VtrA-PL showed that TM domain plays a predominant role in transcriptional activation, implying the oligomerization via the TM domain interactions. We also sought to define the detailed oligomeric state of VtrA in *V*. *parahaemolyticus* cells by the chemical cross-linking using DMA. However, despite considerable experimental efforts, we could not successfully detect any obvious band corresponding to VtrA oligomer in *V*. *parahaemolyticus* at this stage. It is thus uncertain about the detailed oligomeric state of VtrA *in vivo*, and this issue requires further studies for resolution.

*V*. *cholerae* ToxR interacts with the downstream, co-transcribed protein ToxS, which is required for the transcriptional regulatory activity of ToxR [36]. ToxS is hypothesized to stabilize ToxR and enhance the dimerization of its periplasmic domain. Recently, Li *et al*. reported that VtrC, encoded downstream of *vtrA*, is required for the transcriptional regulatory activity of VtrA and suggested that VtrC might stabilize VtrA in *V*. *parahaemolyticus* [18]. This feature of VtrA and VtrC is similar to that of *V*. *cholerae* ToxR and ToxS. It was reported that the expression of ToxR activates *ctx* transcription in *E*. *coli* even in the absence of ToxS, although ToxS is required for lower levels of ToxR to activate transcription, which is associated with an increase in the stability of ToxR [36, 37]. Similarly, we observed that the P_BAD_-based expression of VtrA could activate the P_*vtrB*_*-lacZ* transcription in *E*. *coli*, even without VtrC. Given that the expression of VtrA in *E*. *coli* was relatively overexpression base on the P_BAD_ promoter, it is conceivable that VtrA activates transcription even without VtrC in *E*. *coli*, as is the case with ToxR. This further supports the functional parallel between VtrA /VtrC and ToxR /ToxS.

Bile is a host-derived factor that strongly induces the expression of the Vp-PAI genes of *V*. *parahaemolyticus*, and its stimulation is thought to be responsible for the virulence of *V*. *parahaemolyticus* during intestinal infection. It was recently reported that the periplasmic domain of VtrC and the C-terminal domain of VtrA form a complex where VtrC recruits structural elements from VtrA to complete a β-barrel with a hydrophobic inner chamber that binds bile salts [18]. In this study, we observed that the C-terminal putative periplasmic domain of VtrA was necessary for the bile-induced *vtrB* transcriptional activation, supporting that the C-terminal domain of VtrA participates in sensing bile. Given that oligomerization of VtrA induced its transcriptional regulatory activity, our observation that bile induces VtrA oligomerization could explain the mechanism by which the transcriptional activity of VtrA is activated without affecting its protein levels. In *V*. *cholerae*, the periplasmic domain of ToxR is required for *leuO* and *ompU* upregulation in response to bile, implicating the periplasmic domain of ToxR in sensing environmental stimuli [38]. Bile also stimulates *V*. *cholerae* TcpP transcriptional regulatory activity by mediating intermolecular disulfide bond formation between its periplasmic domains [39]. *E*. *coli* CadC is also known to form an intramolecular disulfide bond within its periplasmic domain, which is important in the function of CadC [40]. However, unlike these ToxR-like proteins, VtrA lacks cysteine residues in its C-terminal domain. Otherwise, bile might affect the conformation of the C-terminal domain of VtrA, bringing the adjacent TM and cytoplasmic DNA-binding domains into close proximity, thus facilitating the oligomerization and DNA-binding activity of VtrA.

The promoter region of *vtrB* contained repetitive 8-bp T-rich DNA elements near the putative −35 promoter element, which are essential for *vtrB* transcriptional activation by VtrA. Interestingly, the VtrA-mediated transcriptional activation of *vtrB* depended on the number of repetitive elements in the *vtrB* promoter region. OmpR-family proteins generally recognize direct repeats in the promoter regions of their target genes through dimerization, allowing these DNA-binding proteins to bind DNA repeats efficiently [41, 42]. Although the precise VtrA-binding site within the *vtrB* promoter region is not yet known, it might be possible that the oligomerization of VtrA promotes the binding to these repetitive DNA elements within the *vtrB* promoter region. It has been proposed that T3SS2-possessing *Vibrio* species may have horizontally acquired the T3SS2α or T3SS2β gene clusters during their evolution [13, 14]. The repetitive DNA elements in the *vtrB* promoter region were also found in T3SS2β-positive *V*. *parahaemolyticus*, T3SS2α-positive *Vibrio cholerae* and T3SS2β-positive *Vibrio cholerae*. Our β-galactosidase assays revealed that VtrA shows *vtrB* transcriptional activation in T3SS2β-positive *V*. *parahaemolyticus* and T3SS2-positive *Vibrio cholerae* strains, suggesting that the mechanism of target recognition by VtrA is also evolutionarily conserved among T3SS2-possessing *Vibrio* species.

In conclusion, we have presented experimental evidence showing that VtrA is a membrane-bound regulator and is active as oligomers. This provides new insights into the mechanism by which VtrA transcriptional regulatory activity is activated, including in response to bile. Further studies will be needed to explore the molecular mechanisms of VtrA oligomerization in detail.

## Supporting information

S1 Fig FLAG-tagged VtrA is localized in the membrane of *V*. *parahaemolyticus* in a TM-dependent manner. Subcellular localization of C-terminal 3×FLAG-tagged VtrA (VtrA-FLAG) and its truncated forms in *V*. *parahaemolyticus*. *V*. *parahaemolyticus* expressing VtrA-FLAG and its truncated derivatives were fractionated into cytosolic (C) and membrane (M) fractions. Total cell lysates (T) and each fraction were subjected to immunoblot analysis for FLAG to detect VtrA and its truncated forms. DnaK and OmpA were detected as controls for the cytosol and membrane, respectively.
(TIF)

S2 Fig VtrA-PL does not activate transcription of *vtrB*. (A) Schematic representations of VtrA and VtrA containing a polyleucine TM domain (VtrA-PL). (B) β-galactosidase activity from the P_*vtrB*_-*lacZ* transcriptional reporter of and *E*. *coli* MC4100 upon expression of VtrA or VtrA-PL. The values represent the mean ±SD for a minimum of three independent experiments.
(TIF)

S3 Fig Oligomeric properties of VtrA^N^-ZIP fusion proteins. Each of the His-tagged VtrA^N^, VtrA^N^-ZIP, VtrA^N^-ZIP^PLI^ and VtrA^N^-ZIP^m^ proteins was treated (+) with dimethyl adipimidate (DMA) or was untreated (−), separated by SDS-PAGE and visualized by Coomassie brilliant blue staining. The migration positions of the molecular weight markers are indicated on the left side of the panel.
(TIF)

S4 Fig His-VtrA^N^ binds to the promoter region of *vtrB* at higher concentrations. Electrophoretic mobility shift assays using VtrA^N^ at concentrations higher than those shown in Fig 3B. Indicated concentrations of His VtrA^N^ were incubated with 4 nM biotinylated DNA probe corresponding to a 284-bp upstream region of *vtrB*. The DNA probe was detected using HRP-conjugated streptavidin.
(TIF)

S5 Fig VtrA activates the *vtrB* promoters of *V*. *parahaemolyticus* T3SS2β-positive strain and *V*. *cholerae* strains possessing T3SS2α and T3SS2β. (A) ClustalW [1] multiple sequence alignments of the upstream sequences of *vtrB* from *V*. *parahaemolyticus* strain RIMD2210633 (Vp_2210633; T3SS2α-positive), *V*. *parahaemolyticus* strain TH3996 (Vp_TH3996; T3SS2β-positive), *V*. *cholerae* strain RIMD2214243 (Vc_2214234; T3SS2α-positive) and *V*. *cholerae* strain RIMD2214428 (Vc_2214428; T3SS2β-positive). Repetitive T-rich elements are indicated by gray shading. (B) Transcriptional activity of VtrA at the upstream promoter regions of *vtrB* from Vp_TH3996, Vc_2214243 and Vc_2214428 was evaluated by measuring β-galactosidase activity using *lacZ* transcriptional reporter in *E*. *coli* MC4100 carrying an empty vector (white bar) or a VtrA expression plasmid (black bar). Data represent the mean ± SD of a minimum of three independent experiments. *, p < 0.01, by Student’s *t*-test.
(TIF)

S1 Table Strains used in this study. (DOCX)

S2 Table Plasmids used in this study. (DOCX)

S1 References (DOCX)

## References

[1] Thompson F. L., Iida T., and Swings J.. Biodiversity of vibrios. Microbiol Mol Biol Rev. 2004;68: 403–431. doi: 10.1128/MMBR.68.3.403-431.2004 15353563 10.1128/MMBR.68.3.403-431.2004 PMC515257

[2] Blake PA, Weaver RE, Hollis DG. Diseases of humans (other than cholera) caused by vibrios. Annu Rev Microbiol. 1980;34: 341–367. doi: 10.1146/annurev.mi.34.100180.002013 7002028 10.1146/annurev.mi.34.100180.002013

[3] Daniels NA, MacKinnon L, Bishop R, Altekruse S, Ray B, Hammond RM, et al *Vibrio parahaemolyticus* infections in the United States, 1973–1998. J Infect Dis. 2000;181: 16661–1666. 10.1086/315459 10823766

[4] Nair GB, Ramamurthy T, Bhattacharya SK, Dutta B, Takeda Y, Sack DA. Global dissemination of *Vibrio parahaemolyticus* serotype O3:K6 and its serovariants. Clin Microbiol Rev. 2007;20: 39–48. doi: 10.1128/CMR.00025-06 17223622 10.1128/CMR.00025-06 PMC1797631

[5] Makino K, Oshima K, Kurokawa K, Yokoyama K, Uda T, Tagomori K, et al Genome sequence of *Vibrio parahaemolyticus*: a pathogenic mechanism distinct from *V*. *cholerae*. Lancet 2003;361: 743–749. doi: 10.1016/S0140-6736(03)12659-1 12620739 10.1016/S0140-6736(03)12659-1

[6] Sugiyama T, Iida T, Izutsu K, Park KS, Honda T. Precise region and the character of the pathogenicity island in clinical *Vibrio parahaemolyticus* strains. J Bacteriol. 2008;190: 1835–1837. doi: 10.1128/JB.01293-07 18156272 10.1128/JB.01293-07 PMC2258670

[7] Izutsu K, Kurokawa K, Tashiro K, Kuhara S, Hayashi T, Honda T, et al Comparative genomic analysis using microarray demonstrates a strong correlation between the presence of the 80-kilobase pathogenicity island and pathogenicity in Kanagawa phenomenon-positive *Vibrio parahaemolyticus* strains. Infect Immun. 2008;76: 1016–1023. doi: 10.1128/IAI.01535-07 18195030 10.1128/IAI.01535-07 PMC2258825

[8] Galán JE, Lara-Tejero M, Marlovits TC, Wagner S. Bacterial type III secretion systems: specialized nanomachines for protein delivery into target cells. Annu Rev Microbiol. 2014;68: 415–438. doi: 10.1146/annurev-micro-092412-155725 25002086 10.1146/annurev-micro-092412-155725 PMC4388319

[9] Hiyoshi H, Kodama T, Iida T, Honda T. Contribution of *Vibrio parahaemolyticus* virulence factors to cytotoxicity, enterotoxicity, and lethality in mice. Infect Immun. 2010;78: 1772–1780. doi: 10.1128/IAI.01051-09 20086084 10.1128/IAI.01051-09 PMC2849405

[10] Ritchie JM, Rui H, Zhou X, Iida T, Kodoma T, Ito S, et al Inflammation and disintegration of intestinal villi in an experimental model for *Vibrio parahaemolyticus*-induced diarrhea. PLoS Pathog. 2012;8: e1002593 doi: 10.1371/journal.ppat.1002593 22438811 10.1371/journal.ppat.1002593 PMC3305451

[11] Kodama T, Hiyoshi H, Okada R, Matsuda S, Gotoh K, Iida T. Regulation of *Vibrio parahaemolyticus* T3SS2 gene expression and function of T3SS2 effectors that modulate actin cytoskeleton. Cell Microbiol. 2015;17: 183–190. doi: 10.1111/cmi.12408 25495647 10.1111/cmi.12408

[12] Dziejman M, Serruto D, Tam VC, Sturtevant D, Diraphat P, Faruque SM, et al Genomic characterization of non-O1, non-O139 *Vibrio cholerae* reveals genes for a type III secretion system. Proc Natl Acad Sci USA. 2005;102: 3465–3470. doi: 10.1073/pnas.0409918102 15728357 10.1073/pnas.0409918102 PMC552950

[13] Okada N, Iida T, Park KS, Goto N, Yasunaga T, Hiyoshi H, et al Identification and characterization of a novel type III secretion system in *trh*-positive *Vibrio parahaemolyticus* strain TH3996 reveal genetic lineage and diversity of pathogenic machinery beyond the species level. Infect Immun. 2009;77: 904–913. doi: 10.1128/IAI.01184-08 19075025 10.1128/IAI.01184-08 PMC2632016

[14] Okada N, Matsuda S, Matsuyama J, Park KS, de los Reyes C, Kogure K, et al Presence of genes for type III secretion system 2 in *Vibrio mimicus* strains. BMC Microbiol. 2010;10: 302 21110901 10.1186/1471-2180-10-302 PMC3004890

[15] Shin OS, Tam VC, Suzuki M, Ritchie JM, Bronson RT, Waldor MK, et al Type III secretion is essential for the rapidly fatal diarrheal disease caused by non-O1, non-O139 *Vibrio cholerae*. MBio 2011;2: e00106–11. doi: 10.1128/mBio.00106-11 21673189 10.1128/mBio.00106-11 PMC3111608

[16] Kodama T, Gotoh K, Hiyoshi H, Morita M, Izutsu K, Akeda Y, et al Two regulators of *Vibrio parahaemolyticus* play important roles in enterotoxicity by controlling the expression of genes in the Vp-PAI region. PLoS One 2010;5: e8678 doi: 10.1371/journal.pone.0008678 20084267 10.1371/journal.pone.0008678 PMC2800187

[17] Gotoh K, Kodama T, Hiyoshi H, Izutsu K, Park KS, Dryselius R, et al Bile acid-induced virulence gene expression of *Vibrio parahaemolyticus* reveals a novel therapeutic potential for bile acid sequestrants. PLoS One 2010;5: e13365 doi: 10.1371/journal.pone.0013365 20967223 10.1371/journal.pone.0013365 PMC2954181

[18] Li P, Rivera-Cancel G, Kinch LN, Salomon D, Tomchick DR, Grishin NV, et al Bile salt receptor complex activates a pathogenic type III secretion system. Elife 2016;5: e15718 doi: 10.7554/eLife.15718 27377244 10.7554/eLife.15718 PMC4933562

[19] Kodama T, Rokuda M, Park KS, Cantarelli VV, Matsuda S, Iida T, et al Identification and characterization of VopT, a novel ADP-ribosyltransferase effector protein secreted via the *Vibrio parahaemolyticus* type III secretion system 2. Cell Microbiol. 2007;9: 2598–2609. doi: 10.1111/j.1462-5822.2007.00980.x 17645751 10.1111/j.1462-5822.2007.00980.x

[20] Miller JH. Experiments in molecular genetics. Cold Spring Harbor Laboratory, Cold Spring Harbor, NY; 1972 pp. 352–355.

[21] Matsuda S, Okada N, Kodama T, Honda T, Iida T. A cytotoxic type III secretion effector of *Vibrio parahaemolyticus* targets vacuolar H^+^-ATPase subunit c and ruptures host cell lysosomes. PLoS Pathog 2012;8: e1002803 doi: 10.1371/journal.ppat.1002803 22829766 10.1371/journal.ppat.1002803 PMC3400558

[22] van Hijum SA, Medema MH, Kuipers OP. Mechanisms and evolution of control logic in prokaryotic transcriptional regulation. Microbiol Mol Biol Rev. 2009;73: 481–509. doi: 10.1128/MMBR.00037-08 19721087 10.1128/MMBR.00037-08 PMC2738135

[23] Ottemann KM, Mekalanos JJ. The ToxR protein of *Vibrio cholerae* forms homodimers and heterodimers. J Bacteriol. 1996; 178: 156*–*162. 8550410 10.1128/jb.178.1.156-162.1996 PMC177633

[24] Gauntlett JC, Gebhard S, Keis S, Manson JM, Pos KM, Cook GM. Molecular analysis of BcrR, a membrane-bound bacitracin sensor and DNA-binding protein from *Enterococcus faecalis*. J Biol Chem. 2008;283: 8591–8600. doi: 10.1074/jbc.M709503200 18227063 10.1074/jbc.M709503200

[25] Goss TJ, Seaborn CP, Gray MD, Krukonis ES. Identification of the TcpP-binding site in the *toxT* promoter of *Vibrio cholerae* and the role of ToxR in TcpP-mediated activation. Infect Immun. 2010;78: 4122–4133. doi: 10.1128/IAI.00566-10 20679441 10.1128/IAI.00566-10 PMC2950353

[26] Dalia AB, Lazinski DW, Camilli A. Identification of a membrane-bound transcriptional regulator that links chitin and natural competence in *Vibrio cholerae*. mBio 2014;5: e01028–13. doi: 10.1128/mBio.01028-13 24473132 10.1128/mBio.01028-13 PMC3903286

[27] Lindner E, White SH. Topology, dimerization, and stability of the single-span membrane protein CadC. J Mol Biol. 2014;426: 2942–2957. doi: 10.1016/j.jmb.2014.06.006 24946151 10.1016/j.jmb.2014.06.006 PMC4126671

[28] Zhou FX, Merianos HJ, Brunger AT, Engelman DM. Polar residues drive association of polyleucine transmembrane helices. Proc Natl Acad Sci USA. 2001;98:2250–2255. doi: 10.1073/pnas.041593698 11226225 10.1073/pnas.041593698 PMC30124

[29] Harbury PB, Zhang T, Kim PS, Alber T. A switch between two-, three-, and four-stranded coiled coils in GCN4 leucine zipper mutants. Science 1993;262: 1401*–*1407. 8248779 10.1126/science.8248779

[30] Fink A, Sal-Man N, Gerber D, Shai Y. Transmembrane domains interactions within the membrane milieu: principles, advances and challenges. Biochem Biophys Acta. 2012;1818: 974–983. doi: 10.1016/j.bbamem.2011.11.029 22155642 10.1016/j.bbamem.2011.11.029

[31] Miller VL, Taylor RK, Makalanos JJ. Cholera toxin transcriptional activator ToxR is a transmembrane DNA binding protein. Cell 1987;48: 271*–*279. 3802195 10.1016/0092-8674(87)90430-2

[32] Goss TJ, Morgan SJ, French EL, Krukonis ES. ToxR recognizes a direct repeat element in the *toxT*, *ompU*, *ompT*, and *ctxA* promoters of *Vibrio cholerae* to regulate transcription. Infect Immun. 2013;81: 884–895. doi: 10.1128/IAI.00889-12 23297386 10.1128/IAI.00889-12 PMC3584884

[33] Lin Z, Kumagai K, Baba K, Mekalanos JJ, Nishibuchi M. *Vibrio parahaemolyticus* has a homolog of the *Vibrio cholerae* toxRS operon that mediates environmentally induced regulation of the thermostable direct hemolysin gene. J Bacteriol. 1993;175: 3844–3855. 8509337 10.1128/jb.175.12.3844-3855.1993 PMC204801

[34] Kenney LJ. Structure/function relationships in OmpR and other winged-helix transcription factors. Curr Opin Microbiol. 2002;5: 135–141. 11934608 10.1016/s1369-5274(02)00310-7

[35] Bajaj V, Hwang C, Lee CA. *hilA* is a novel *ompR*/*toxR* family member that activates the expression of *Salmonella typhimurium* invasion genes. Mol Microbiol. 1995;18: 715–727. doi: 10.1111/j.1365-2958.1995.mmi_18040715.x 8817493 10.1111/j.1365-2958.1995.mmi_18040715.x

[36] DiRita VJ, Mekalanos JJ. Periplasmic interaction between two membrane regulatory proteins, ToxR and ToxS, results in signal transduction and transcriptional activation. Cell 1991;64: 29*–*37. 1898871 10.1016/0092-8674(91)90206-e

[37] Miller VL, DiRita VJ, Mekalanos JJ. Identification of *toxS*, a regulatory gene whose product enhances ToxR-mediated activation of the cholera toxin promoter. J. Bacteriol. 1989;171:1288*–*1293. 2646275 10.1128/jb.171.3.1288-1293.1989 PMC209743

[38] Ante VM, Bina XR, Howard MF, Sayeed S, Taylor DL, Bina JE. *Vibrio cholerae leuO* transcription is positively regulated by ToxR and contributes to bile resistance. J Bacteriol. 2015;197: 3499–3510. doi: 10.1128/JB.00419-15 26303831 10.1128/JB.00419-15 PMC4621094

[39] Yang M, Liu Z, Hughes C, Stern AM, Wang H, Zhong Z, et al Bile salt-induced intermolecular disulfide bond formation activates *Vibrio cholerae* virulence. Proc Natl Acad Sci USA. 2013;110: 2348–2353. doi: 10.1073/pnas.1218039110 23341592 10.1073/pnas.1218039110 PMC3568309

[40] Tetsch L, Koller C, Dönhöfer A, Jung K. Detection and function of an intramolecular disulfide bond in the pH-responsive CadC of *Escherichia coli*. BMC Microbiol. 2011;11: 74 doi: 10.1186/1471-2180-11-74 21486484 10.1186/1471-2180-11-74 PMC3096576

[41] Martínez-Hackert E, Stock AM. Structural relationships in the OmpR family of winged-helix transcription factors. J Mol Biol. 1997;269: 301*–*312. doi: 10.1006/jmbi.1997.1065 9199401 10.1006/jmbi.1997.1065

[42] Blanco AG, Sola M, Gomis-Rüth FX, Coll M. Tandem DNA recognition by PhoB, a two-component signal transduction transcriptional activator. Structure 2002;10: 701*–*713. 12015152 10.1016/s0969-2126(02)00761-x

