# Supplementary figures and images for: Vibrio parahaemolyticus VtrA is a membrane-bound regulator and is activated via oligomerization
Source: PLoS One. 2017 Nov 17;12(11):e0187846. doi: 10.1371/journal.pone.0187846 (PMC5693285; doi:10.1371/journal.pone.0187846)

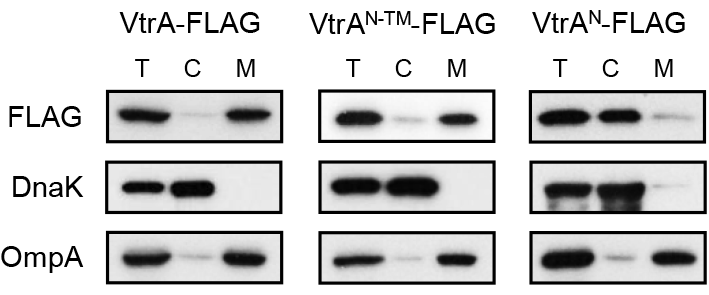

Supplement: S1 Fig — Subcellular localization of C-terminal 3×FLAG-tagged VtrA (VtrA-FLAG) and its truncated forms in V. parahaemolyticus. V. parahaemolyticus expressing VtrA-FLAG and its truncated derivatives were fractionated into cytosolic (C) and membrane (M) fractions. Total cell lysates (T) and each fraction were subjected to immunoblot analysis for FLAG to detect VtrA and its truncated forms. DnaK and OmpA were detected as controls for the cytosol and membrane, respectively. (TIF) [file pone.0187846.s001.tif]

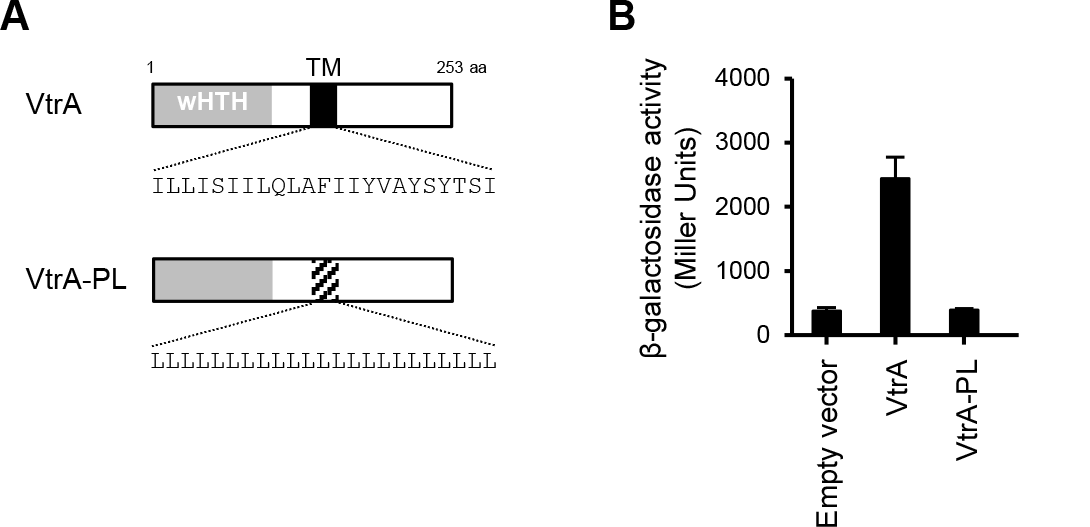

Supplement: S2 Fig — (A) Schematic representations of VtrA and VtrA containing a polyleucine TM domain (VtrA-PL). (B) β-galactosidase activity from the PvtrB-lacZ transcriptional reporter of and E. coli MC4100 upon expression of VtrA or VtrA-PL. The values represent the mean ±SD for a minimum of three independent experiments. (TIF) [file pone.0187846.s002.tif]

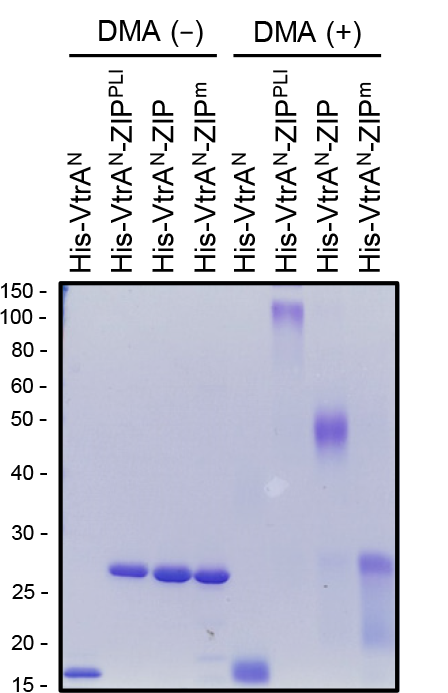

Supplement: S3 Fig — Each of the His-tagged VtrAN, VtrAN-ZIP, VtrAN-ZIPPLI and VtrAN-ZIPm proteins was treated (+) with dimethyl adipimidate (DMA) or was untreated (−), separated by SDS-PAGE and visualized by Coomassie brilliant blue staining. The migration positions of the molecular weight markers are indicated on the left side of the panel. (TIF) [file pone.0187846.s003.tif]

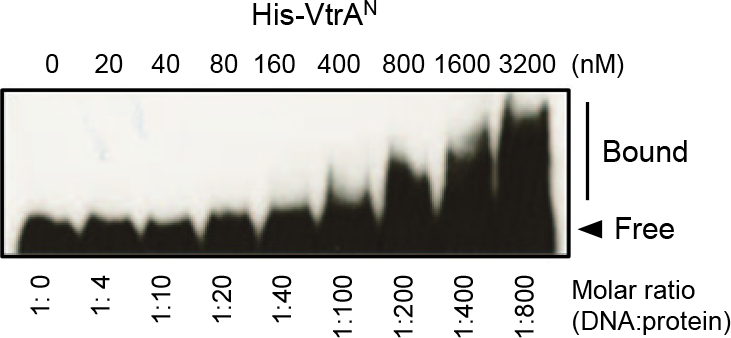

Supplement: S4 Fig — Electrophoretic mobility shift assays using VtrAN at concentrations higher than those shown in Fig 3B. Indicated concentrations of His VtrAN were incubated with 4 nM biotinylated DNA probe corresponding to a 284-bp upstream region of vtrB. The DNA probe was detected using HRP-conjugated streptavidin. (TIF) [file pone.0187846.s004.tif]

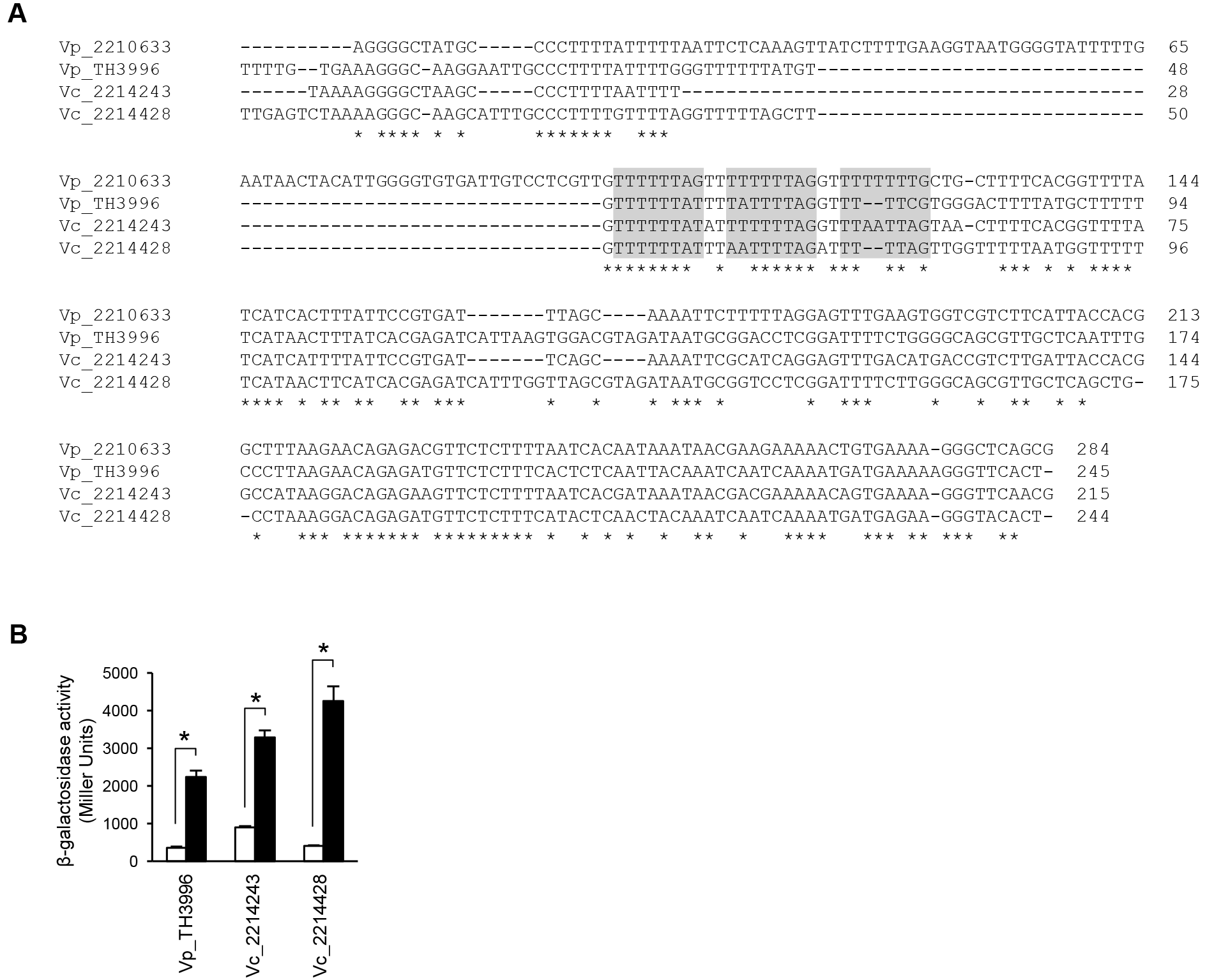

Supplement: S5 Fig — (A) ClustalW [1] multiple sequence alignments of the upstream sequences of vtrB from V. parahaemolyticus strain RIMD2210633 (Vp_2210633; T3SS2α-positive), V. parahaemolyticus strain TH3996 (Vp_TH3996; T3SS2β-positive), V. cholerae strain RIMD2214243 (Vc_2214234; T3SS2α-positive) and V. cholerae strain RIMD2214428 (Vc_2214428; T3SS2β-positive). Repetitive T-rich elements are indicated by gray shading. (B) Transcriptional activity of VtrA at the upstream promoter regions of vtrB from Vp_TH3996, Vc_2214243 and Vc_2214428 was evaluated by measuring β-galactosidase activity using lacZ transcriptional reporter in E. coli MC4100 carrying an empty vector (white bar) or a VtrA expression plasmid (black bar). Data represent the mean ± SD of a minimum of three independent experiments. *, p < 0.01, by Student’s t-test. (TIF) [file pone.0187846.s005.tif]
