# Supplementary material for: Vibrio parahaemolyticus VtrA is a membrane-bound regulator and is activated via oligomerization
Source: PLoS One. 2017 Nov 17;12(11):e0187846. doi: 10.1371/journal.pone.0187846 (PMC5693285; doi:10.1371/journal.pone.0187846)
Supplement: S1 Table — (DOCX) [file pone.0187846.s006.docx]

| **Strain** | **Genotypes/Descriptions** | **References** |
| --- | --- | --- |
| *V. parahaemolyticus* |  |  |
| RIMD2210633 | a T3SS2α-positive clinical isolate; wild-type | [2] |
| Δ*vtrA* | *vtrA*; stop codon at aa 30 and deletion from nucleotides 88 to 471 of the gene | [3] |
| Δ*vtrA* Δ*vp0820* | *vtrA vp0820* | This study |
| POR-2Δ*vtrA* | *tdhA tdhS vcrD1 vtrA*; TDH-, T3SS1- and VtrA-deficient | [3] |
| POR-2Δ*vcrD2* | *tdhA tdhS vcrD1 vcrD2*; TDH-, T3SS1- and T3SS2-defective | [3] |
| TH3996 | a T3SS2β-positive clinical isolate | [4] |
| *V. cholerae* |  |  |
| RIMD2214243 | a clinical isolate, serotype O5, T3SS2α-positive | [4] |
| RIMD2214428 | a clinical isolate, serotype O190, T3SS2β-positive | [5] |
| *E. coli* |  |  |
| DH5α | F^−^ Φ80Δ*lacZ*M15 Δ(*lacZYA argF*)U169 *deoP recA1 endA1 hsdR17*(rK^−^ mK^−^) | Laboratory collection |
| SM10λpir | *thi thr leu tonA lacY supE recA*::RP4-2-Tc::Mu λ*pir* R6K | [6] |
| MC4100 | F^−^ *araD139* Δ(*argF-lac*) *U169 rpsL150* (Str^r^) *relA1 flbB5301 deoC1 ptsF25 rbsR* | [7] |
|  |  |  |
|  |  |  |
