# Supplementary material for: Vibrio parahaemolyticus VtrA is a membrane-bound regulator and is activated via oligomerization
Source: PLoS One. 2017 Nov 17;12(11):e0187846. doi: 10.1371/journal.pone.0187846 (PMC5693285; doi:10.1371/journal.pone.0187846)
Supplement: S2 Table — (DOCX) [file pone.0187846.s007.docx]

| **Plasmid** | **Descriptions** | **References** |
| --- | --- | --- |
| pBAD18-Cm | P_BAD_ promoter, pBR322 *ori*, Cm^R^ | [8] |
| pBAD-*vtrA* | pBAD18-Cm carrying *HA-vtrA* | This study |
| pBAD-*vtrA^N-TM^* | pBAD18-Cm carrying *HA-vtrA* (1–156) | This study |
| pBAD-*vtrA^N^* | pBAD18-Cm carrying *HA-vtrA* (1–133) | This study |
| pBAD- *vtrA*-PL | pBAD18-Cm carrying *HA-vtrA* containing the polyleucine TM domain | This study |
| pBAD-*vtrA-FLAG* | pBAD18-Cm carrying *vtrA-3×FLAG* | This study |
| pBAD-*vtrA^N-TM^-FLAG* | pBAD18-Cm carrying *vtrA*(1–156)*-3×FLAG* | This study |
| pBAD-*vtrA^N^-FLAG* | pBAD18-Cm carrying *vtrA* (1–133)*-3×FLAG* | This study |
| pBAD-*vtrA^N^-ZIP* | pBAD18-Cm carrying *vtrA-ZIP-3×FLAG* | This study |
| pBAD-*vtrA^N^-ZIP^PLI^* | pBAD18-Cm carrying *vrA* (1–133)*-ZIP^PLI^-3×FLAG* | This study |
| pBAD-*vtrA^N^-ZIP^m^* | pBAD18-Cm carrying *vtrA* (1–133)*-ZIP^m^-3×FLAG* | This study |
| pBAD-*toxR* | pBAD18-Cm carrying *toxR_VC_* | This study |
| pBAD-*toxR^N^* | pBAD18-Cm carrying *toxR_VC_* (1–182) | This study |
| pBAD-*toxR^N^-vtrA^TM-C^* | pBAD18-Cm carrying *toxR_VC_* (1–182)*-vtrA* (134-253) | This study |
| pET28a | Expression vector for *E. coli*, Km^R^ | Novagen |
| pET28a-*vtrA^N^* | pET28a carrying *vtrA* (1–133) | This study |
| pET28a-*vtrA^N^-ZIP* | pET28a carrying *vtrA* (1–133)*-ZIP* | This study |
| pET28a-*vtrA^N^-ZIP^PLI^* | pET28a carrying *vtrA*(1–133)*-ZIP^PLI^* | This study |
| pET28a-*vtrA^N^-ZIP^m^* | pET28a carrying *vtrA*(1–133)*-ZIP^m^* | This study |
| pHRP309 | *lacZ* transcriptional fusion vector, Gm^R^ | [9] |
| pHRP309-P*_vtrB_* | pHRP309 containing 284 bp upstream promoter region of *vtrB* | [3] |
| pHRP309-P*_ompU_* | pHRP309 containing promoter region of *ompU_VC_*　(−211 to +22) | This study |
| pHRP309-up*_vtrB_* (−87) | pHRP309 containing 189 bp upstream promoter region of *vtrB* | This study |
| pHRP309-up*_vtrB_* (−78) | pHRP309 containing 180 bp upstream promoter region of *vtrB* | This study |
| pHRP309-up*_vtrB_* (−68) | pHRP309 containing 170 bp upstream promoter region of *vtrB* | This study |
| pHRP309-up*_vtrB_* (−58) | pHRP309 containing 160 bp upstream promoter region of *vtrB* | This study |
| pHRP309-up*_vtrB_* (−48) | pHRP309 containing 150 bp upstream promoter region of *vtrB* | This study |
| pHRP309 -P*_vtrB_* (ΔTRE) | pHRP309 containing promoter region of *vtrB* in which T-rich elements are deleted | This study |
| pHRP309-P*_vtrB_* _(TH3996)_ | pHRP309 containing 245 bp upstream promoter region of *vtrB_TH3996_* | This study |
| pHRP309-P*_vtrB_* _(Vc_2214243)_ | pHRP309 containing 214 bp upstream promoter region of *vtrB_VC2214243_* | This study |
| pHRP309-P*_vtrB_* _(Vc_2214428)_ | pHRP309 containing 244 bp upstream promoter region of *vtrB_VC2214428_* | This study |
