# Supplementary material for: Vibrio parahaemolyticus VtrA is a membrane-bound regulator and is activated via oligomerization
Source: PLoS One. 2017 Nov 17;12(11):e0187846. doi: 10.1371/journal.pone.0187846 (PMC5693285; doi:10.1371/journal.pone.0187846)
Supplement: S1 References — (DOCX) [file pone.0187846.s008.docx]

**References for Supporting Information**

1. Thompson JD, Higgins DG, Gibson TJ. CLUSTAL W: improving the sensitivity of progressive multiple sequence alignment through sequence weighting, position-specific gap penalties and weight matrix choice. Nucleic Acids Res 1994;22: 4673–4680.
2. Makino K, Oshima K, Kurokawa K, Yokoyama K, Uda T, Tagomori K, et al. Genome sequence of *Vibrio parahaemolyticus*: a pathogenic mechanism distinct from *V. cholerae*. Lancet 2003;361: 743–749.
3. Kodama T, Gotoh K, Hiyoshi H, Morita M, Izutsu K, Akeda Y, et al. Two regulators of Vibrio parahaemolyticus play important roles in enterotoxicity by controlling the expression of genes in the Vp-PAI region. PLoS One 2010;5: e8678.
4. Okada N, Iida T, Park KS, Goto N, Yasunaga T, Hiyoshi H, et al. Identification and characterization of a novel type III secretion system in *trh*-positive Vibrio parahaemolyticus strain TH3996 reveal genetic lineage and diversity of pathogenic machinery beyond the species level. Infect Immun. 2009;77: 904–913.
5. Okada N, Matsuda S, Matsuyama J, Park KS, de los Reyes C, Kogure K, et al. Presence of genes for type III secretion system 2 in *Vibrio mimicus* strains. BMC Microbiol. 2010;10: 302.
6. Miller VL, Mekalanos JJ. A novel suicide vector and its use in construction of insertion mutations: osmoregulation of outer membrane proteins and virulence determinants in *Vibrio cholerae* requires *toxR*. J Bacteriol 1988;170: 2575–2583
7. Casadaban MJ. Transposition and fusion of the lac genes to selected promoters in Escherichia coli using bacteriophage lambda and Mu. J Mol Biol 1976;104: 541–555.
8. Guzman LM, Belin D, Carson MJ, Beckwith J. Tight regulation, modulation, and high-level expression by vectors containing the arabinose P_BAD_ promoter. J Bacteriol 1995;177: 4121–4130.
9. Parales RE, Harwood CS. Construction and use of a new broad-host-range *lacZ* transcriptional fusion vector, pHRP309, for Gram^−^ bacteria. Gene 1993;133: 23–30.
